# Investigation of a Miniaturized Four-Element Antenna Integrated with Dipole Elements and Meta-Couplers for 5G Applications

**DOI:** 10.3390/s22145335

**Published:** 2022-07-17

**Authors:** Asutosh Mohanty, Bikash Ranjan Behera, Nasimuddin Nasimuddin, Mohammed H. Alsharif, Peerapong Uthansakul, Syed Agha Hassnain Mohsan

**Affiliations:** 1 School of Electronics Engineering, Kalinga Institute of Industrial Technology Bhubaneswar, Bhubaneswar 751024, India; asutoshmohanty.kiit0409@gmail.com; 2 Department of Electronics and Communication Engineering, Centre for Advanced Post Graduate Studies (BPUT) Rourkela, Rourkela 769015, India; bikashranjan.behera@gmail.com; 3 Institute for Infocomm Research, Singapore 138632, Singapore; nasimuddin@i2r.a-star.edu.sg; 4 Department of Electrical Engineering, College of Electronics and Information Engineering, Sejong University, Seoul 05006, Korea; 5 School of Telecommunication Engineering, Suranaree University of Technology, Nakhon Ratchasima 30000, Thailand; 6 Ocean College, Zhejiang University, Zheda Road 1, Zhoushan 316021, China; hassnainagha@zju.edu.cn

**Keywords:** four-element antenna, dipole elements, complementary split ring resonator (CSRR), meta-couplers, surface-wave trapments, 5G applications

## Abstract

A miniaturized four-element antenna of 20 mm × 20 mm with edge-to-edge distance of 4.9 mm between the array antennas operating from 4.6–8.6 GHz is investigated in this article. The antenna consists of 4 × integrated dipole driven elements, and complementary split ring resonator (CSRR) metacells are loaded on the both sides of each dipole arms. The loaded meta-couplers magnetically couple to dipole drivers, and the induced resonance effect improves the 10-dB impedance bandwidth (IBW) to 60.6%. To improvise the isolation between antenna elements, metallic vias are implemented that trap electromagnetic (EM)-surface waves to condense into the ground. So, the meta-couplers induce electromagnetic (EM)-propagation as surface wave trapments for radiation and decouple near-field condensed currents, acting as couplers/decouplers. The maximum isolation achieved is >−22.5 dB without any external decoupling network. The diversity parameters indicate good attributes in isotropic, indoor, and outdoor channel environments with an envelope correlation coefficient (ECC) < 0.165 and realized gain of 5.5 dBi with average radiation efficiency of 80–90% in the desired operating bands. An equivalent circuit model using lumped components is designed for the proposed four-element antenna. For validation, a prototype antenna is fabricated and measured to be implemented in 5G applications, which shows good correlation with the full-wave simulated results.

## 1. Introduction

Multi-element antennas play an important role in RF wireless communication architecture [1,2,3,4,5,6,7,8] and its development towards uninterrupted high-speed data transfer with an increased efficiency, higher bandwidth, and low power consumption. With 5G networks [9,10,11] and adoption of the derivatives such as 5G chipsets, the Internet-of-Things (IoTs), Machine-2-Machine (M2M) connectivity, multi-element antennas are integrated to optimize the performance with high bandwidth and low latency. The optimal feature is to synchronize the signal transmission process between Tx and Rx [12,13,14,15,16,17,18,19,20,21,22,23]. This type of transmission process depends on antenna metrics, such as antenna sizes, bandwidth, isolations, and envelope correlation coefficients (ECC). To a certain extent, the frequency selective surfaces (FSSs) help in controlling the MIMO performances [24,25]. The main geometrical challenges in a multi-element antenna system for the RF wireless environment are as follows:

### 1.1. Compact Design Footprint

These multi-element antenna systems are designed with the arrayed metallic components, clusters, and more radiating elements. To design a compact multi-element antenna, the orientation and placement of the radiating elements play a crucial role in attaining the suitable radiation and isolation characteristics [26,27,28]. So, the radiators need to be designed to accommodate to a compact footprint for specific wireless applications [29,30,31].

### 1.2. Efficient Decoupling Network

An efficient decoupling network in a multi-element antenna has low mutual-coupling between the adjacent radiating elements [32,33,34]. The decoupling network should be precisely designed to avoid cross-coupling attributes, which would decrease the efficiency of the antenna system of near-field radiated waves from different conducting elements. The metasurface structures as decouplers have the advantages of miniaturization [35,36,37].

### 1.3. Good Isolation Coefficient

A good isolation coefficient in the case of a multi-element antenna tracks the isolation of a transmitted signal in discrete antenna ports [38,39,40,41,42,43,44,45,46]. The lower the isolation coefficients, the better the matching on the antenna ports with a reduction in the mutual coupling.

### 1.4. Good Diversity Metrics

In general, a good diversity multi-element antenna has pattern or polarization radiation characteristics with a lower correlation coefficient in between the far-field radiated patterns. The diversity parameters show the capability for the spatial multiplexing of correlated/uncorrelated signals in indoor, outdoor, and isotropic channel environments [43,44,45,46,47,48,49,50,51,52].

In viewing of the current requirements for an RF wireless communication system, we propose the miniaturized four-element antenna operating at the wideband mode with good performance. It has no decoupling network and simple dipole antenna elements with a meta-coupler acting as a radiator and decoupler. The integration of metallic vias improves the bandwidth and isolation. Since, the meta-couplers have good EM-surface wave propagation characteristics, they help to achieve miniaturization, which is quite important from the application perspective.

## 2. One-Element Antenna Configuration: Design, Study, and Its Analysis

**Figure 1:**
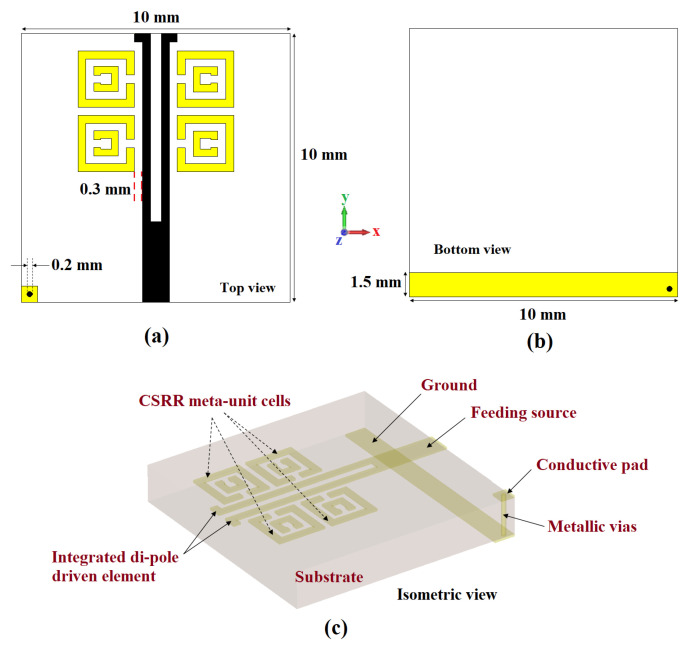
Proposed one-element antenna: (**a**) top view, (**b**) bottom view, and (**c**) isometric view.

**Figure 2:**
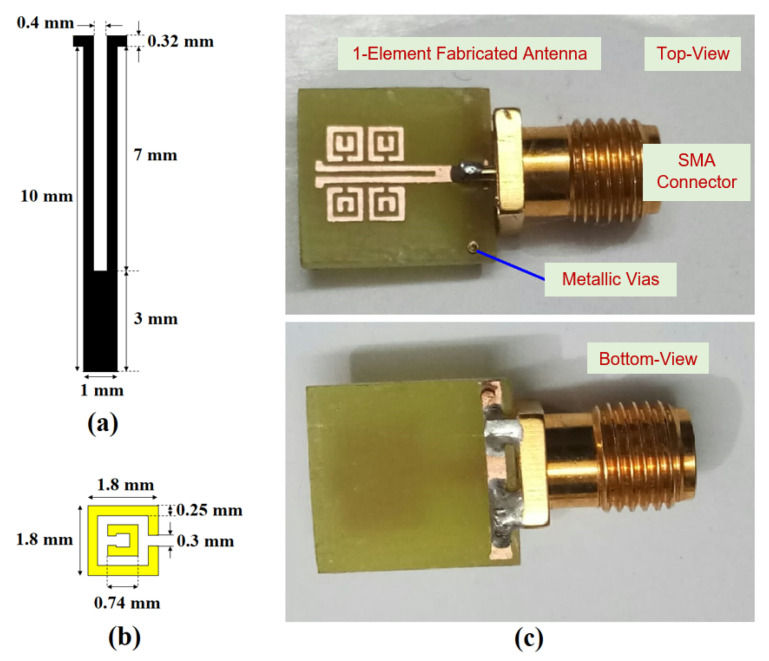
Fabricated prototype with schematics: (**a**) dipole feed, (**b**) meta-coupler, and (**c**) one-element antenna.

**Figure 3:**
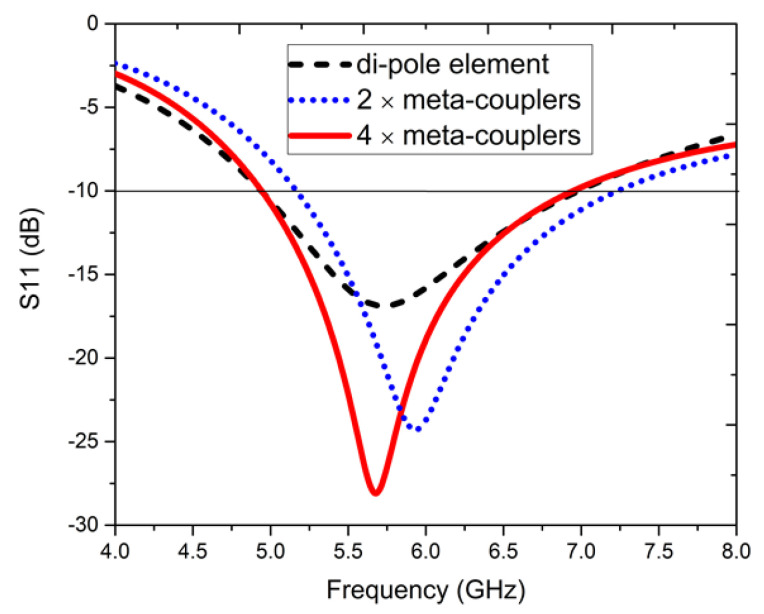
S11, dB parameters of different configurations for the one-element antenna configuration.

**Figure 4:**
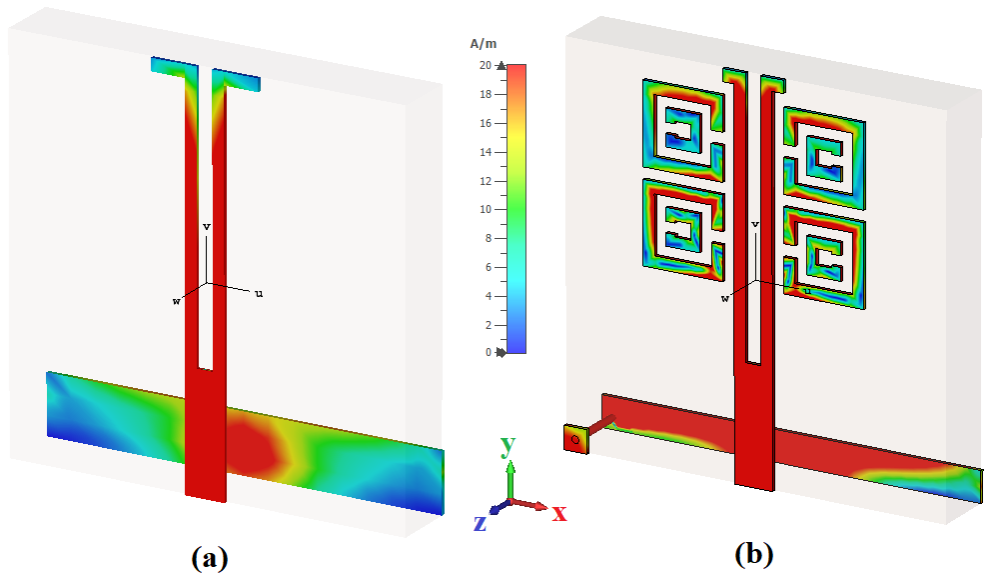
Surface current distributions of the one-element antenna at 5.8 GHz for: (**a**) dipole driven element and (**b**) dipole with meta-coupler.

Here, Figure 1 highlights the top, bottom, and isometric views of a one-element antenna, which was fabricated on an FR4 substrate (thickness = 1.6 mm, ϵ${}_{r}$ = 4.3, and tanδ = 0.02) of size 10 mm × 10 mm. The top layer consists of a dipole driven with 4× meta-couplers, which are inductively coupled, and metallic vias are introduced via a conductive pad and the ground plane [53]. The antenna dimensions in millimeters (mm) are shown in the corresponding figures. Figure 2 shows the cross-sectional view of the dipole element and meta-coupler. The S11, dB parameters for different configurations are shown in Figure 3. It is also observed that with only dipole element, satisfactory matching characteristics are observed; with 2× meta-coupler, the matching is improved, as with the 4× meta-coupler. The impedance surface current distributions at 5.8 GHz are shown in Figure 4 for the dipole element and the proposed one-element antenna configuration with the excitation of signal amplitude from 0 to 20 A/m.

## 3. Four-Element Antenna Configuration: Design, Study, and Its Analysis

**Figure 5:**
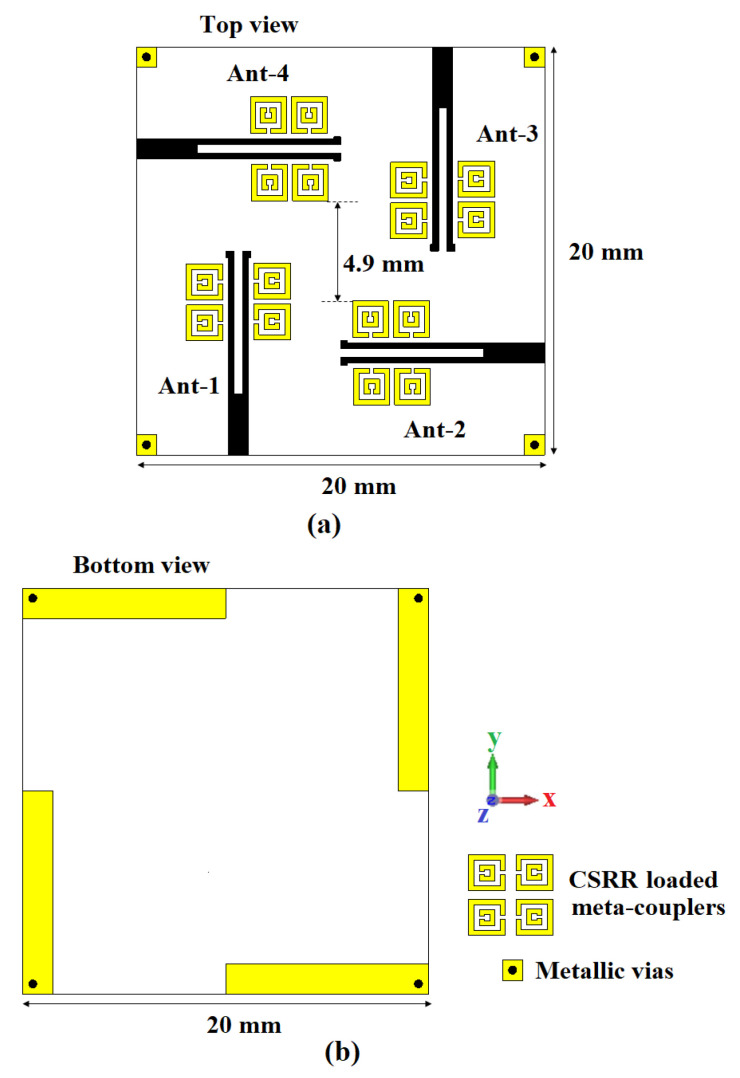
Proposed four-element antenna configuration: (**a**) top view and (**b**) bottom view.

Figure 5 presents the top and bottom views of a four-element antenna configuration which was fabricated on an FR4 substrate (thickness = 1.6 mm, ϵ${}_{r}$ = 4.3, and tanδ = 0.02) of size 20 mm × 20 mm. The top layer consists of orthogonally-oriented dipole driven elements and meta-couplers. The metallic vias are implemented via conductive pads, and the ground is to keep the usual port signals on a common reference plane [54]. In general, the above proposed four-element configuration follows the principles of the splitted ground technique, which is a unique way of attaining improved performances [55,56,57,58]. However, the implementation of vias, in our case, resulted in a significant improvement in the impedance characteristics, which can be seen in the result analysis.

**Figure 6:**
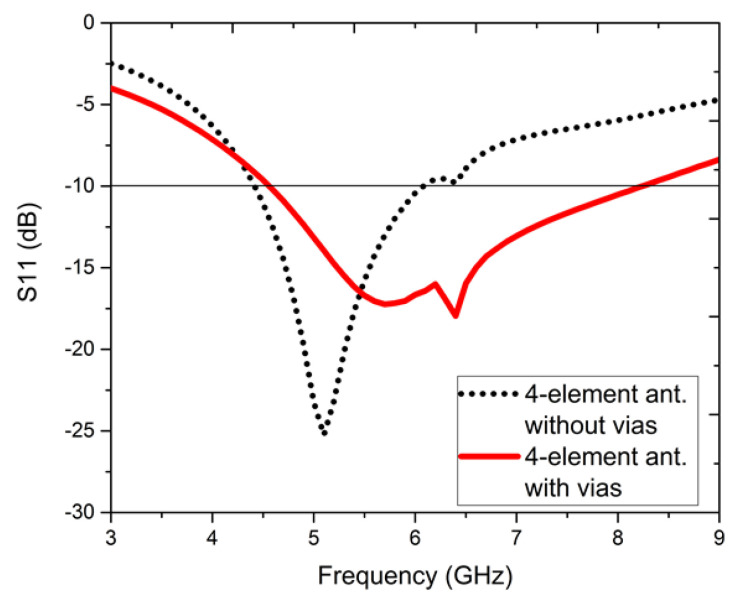
S11, dB parameters without and with vias for the four-element antenna configuration.

Figure 6 presents the S11, dB responses without and with vias, when port-1 is excited and port-2, 3, 4 are impedance-matched with 50-Ω loads. Without vias, the S11, dB varies from 4.5 to 6 GHz with 28.57% IBW and with vias, the S11, dB varies from 4.6 to 8.6 GHz with 60.6% IBW, with an improvement from 28.57% to 60.6%. Hence, the vias not only widen the band but also increase the reflections in port-1, 2, 3, and 4.

### 3.1. Configuration without Vias

**Figure 7:**
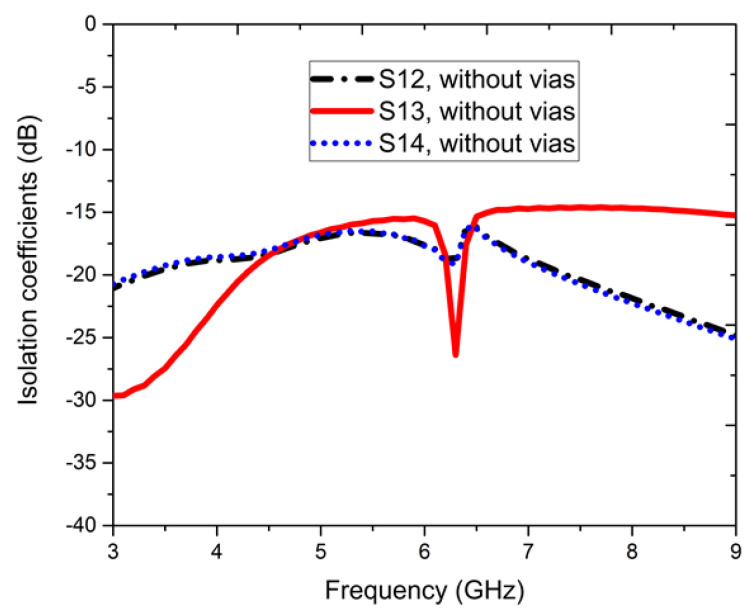
Isolation parameters, dB without vias for the four-element antenna configuration.

**Figure 8:**
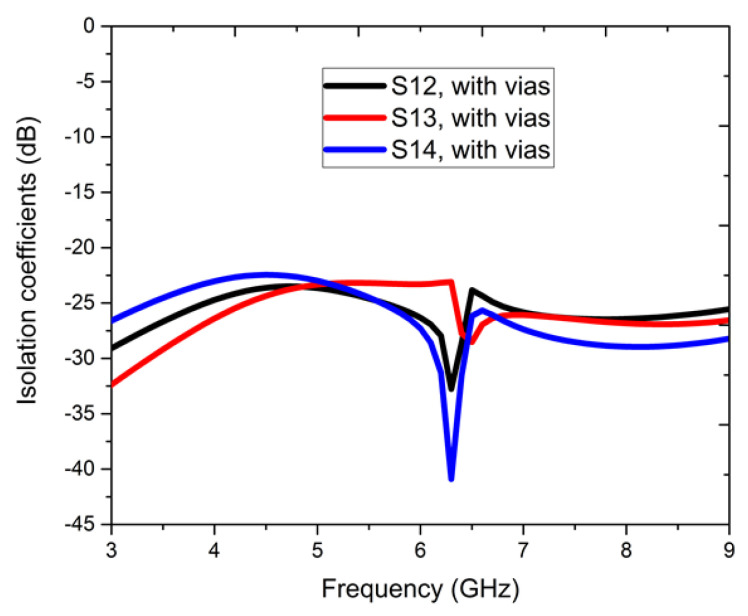
Isolation parameters, dB with vias for the four-element antenna configuration.

**Figure 9:**
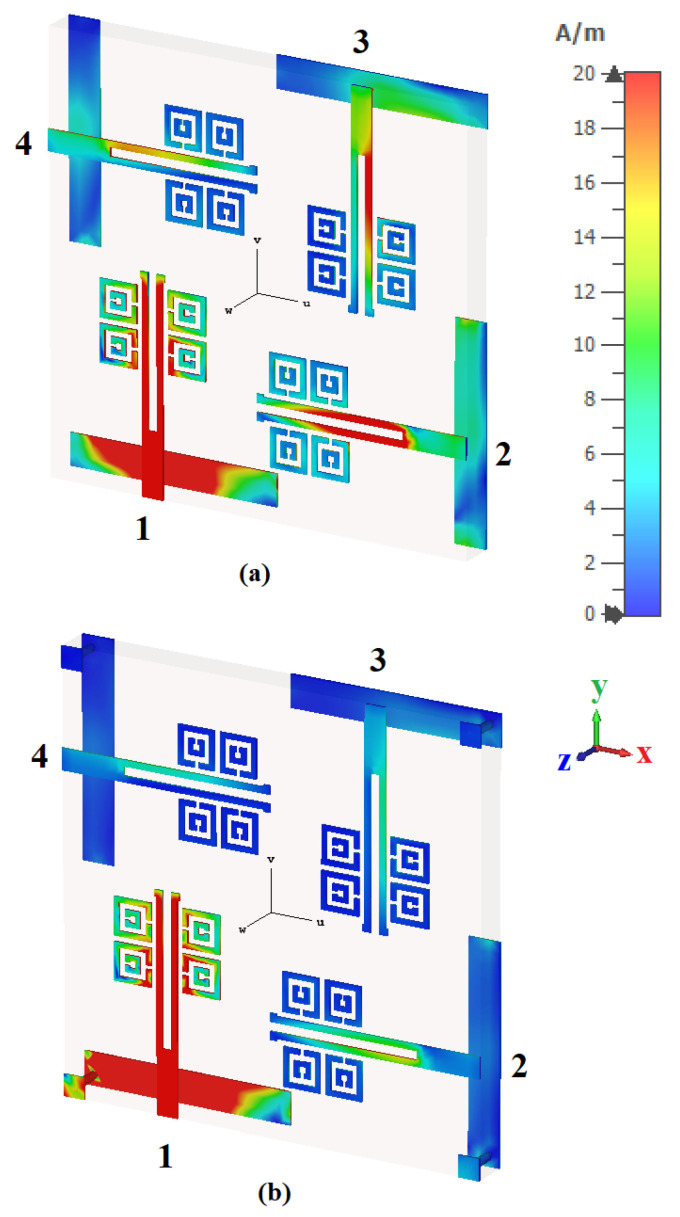
Surface current distributions at 5.8 GHz of the four-element antenna configuration: (**a**) without vias and (**b**) with vias.

Figure 7 presents the isolation coefficients without vias; the S12, S13, and S14 parameters show a magnitude of −15 dB. Similarly, in Figure 8, a response for the isolation coefficients with vias are shown. In Figure 9, it is observed that when port-1 is excited, the currents are reflected at adjacent ports and condensed at the dipole elements of port-2, 3, and 4 with the excitation of signal amplitude from 0 to 20 A/m. Here, the meta-couplers mitigate this by coupling a fraction of the surface waves to be condensed into adjacent ports.

### 3.2. Configuration with Vias

**Figure 10:**
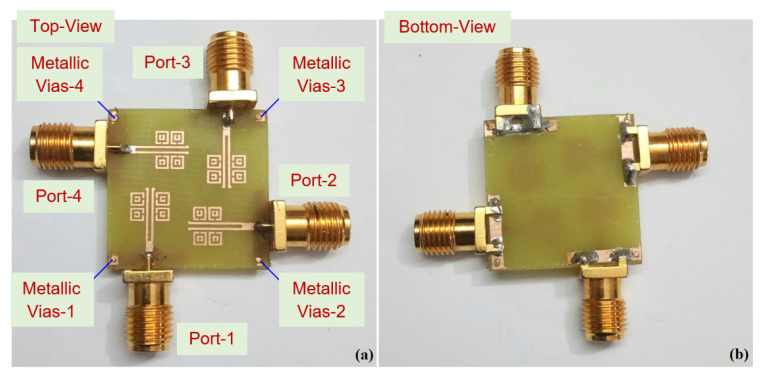
Fabricated prototype of the proposed four-element antenna: (**a**) top view and (**b**) bottom view.

Figure 8 shows the isolation coefficients with vias; the S12, S13, and S14 parameters show a magnitude of >−20 dB, as compared to the attained isolation of −15 dB without vias. In Figure 9, it is observed that when port-1 is excited, the near-field waves radiating are coupled into the meta-couplers, and a large fraction of surface waves are coupled into the ground via the metallic vias, as the self-mitigation process is acknowledged. Hence, the isolation magnitudes are reduced, and the condensed currents present at the adjacent ports are self-mitigated into the respective metallic vias. The proposed four-element antenna configuration avoided the need for a supplementary decoupling network; therefore, the prototype is simple, compact, and performance-centric. Figure 10 shows the fabricated photo of the proposed four-element antenna using the PCB prototyping mechanism (PCB-ETSMATE).

### 3.3. Effects of Metallic Vias and Meta-Couplers

**Figure 11:**
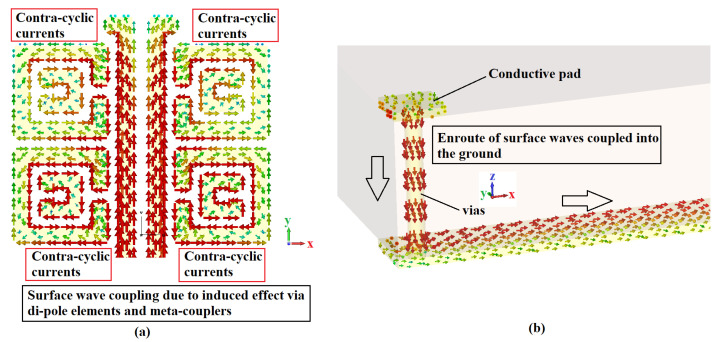
(**a**) Surface wave coupling process and (**b**) metallic vias’ effect on the case of the proposed four-element antenna configuration.

Here, the currents from the dipole elements were induced into the meta-couplers, which are magnetically coupled resulting in a contra-cyclic current loop. It couples a larger amount of EM surface waves for radiation and the self-mitigation of near-field radiated waves. The metallic vias are one-ended cornered to route the surface waves to be coupled into the proposed ground; thus, the mutual-coupling effect from the adjacent ports is self-mitigated. The aforementioned effects are clearly shown in the simulated surface currents, Figure 11a,b, respectively.

## 4. Parametric Study

**Figure 12:**
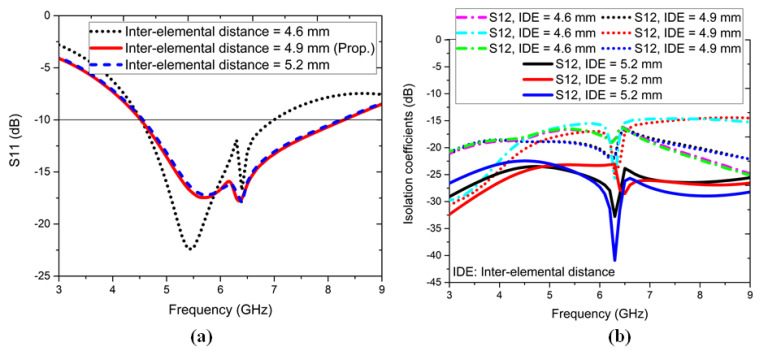
Variation of interelemental distance (IDE): (**a**) S11, dB parameter and (**b**) S12, S13, and S14 dB parameters.

In this section, we describe the variation in the important antenna parameters to attain the optimum scattering and isolation coefficients. Figure 12 shows the effect of S11, dB and the S12, S13, and S14 dB parameters on the variation of the interelemental distance (IDE) between consecutive antenna elements; when IDE = 4.6 mm, the lower band remained constant, and the upper band shrank, but the isolation coefficients were −15 dB. When IDE = 5.2 mm, a good matching was observed, but the isolation coefficients showed a magnitude of −15 dB. The proposed IDE = 4.9 mm had good S11, dB, and the isolation coefficients were >−20 dB.

**Figure 13:**
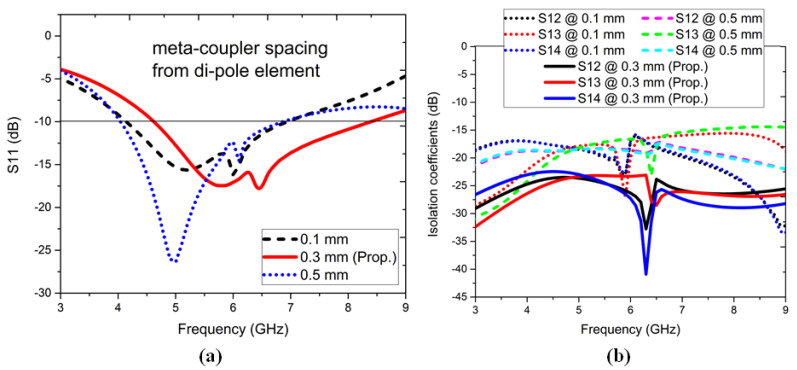
Variation of the distance between the dipole element and meta-coupler: (**a**) S11, dB parameter and (**b**) S12, S13, and S14 dB parameters.

The spacing between the meta-couplers and dipole element was varied, as shown in Figure 13. The S11, dB with a separation value of 0.1 mm showed wideband matching with shifting in the lower bands, but the isolation coefficients showed a magnitude of −15 dB. When the separation value = 0.5 mm, the upper band matching shrank, and the isolation coefficients were still −15 dB. When the separation = 0.3 mm, a good impedance matching was observed with the isolation coefficients > −20 dB in the desired operating frequency bands.

**Figure 14:**
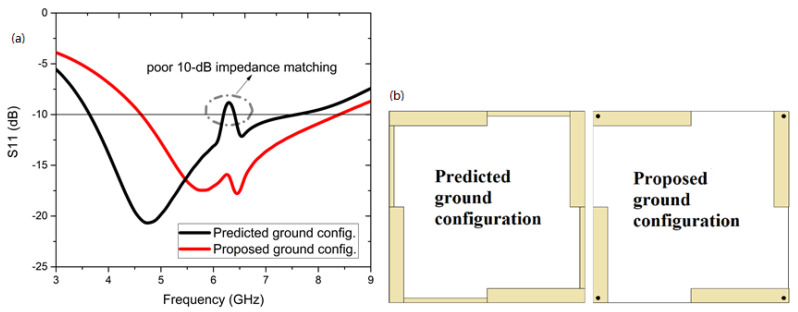
Variation of the ground: (**a**) predicted ground configuration and (**b**) proposed ground configuration.

**Figure 15:**
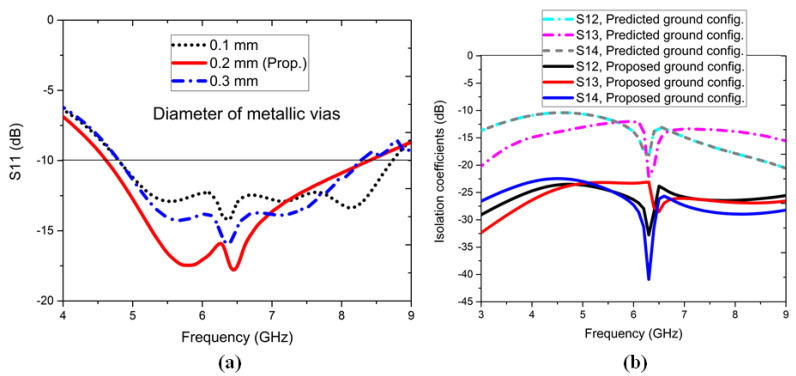
(**a**) Effect of S11, dB on vias’ diameters and (**b**) isolation coefficients of the predicted ground configuration and proposed ground configuration.

Figure 14 highlights the S11, dB plots for the predicted and proposed antenna configurations. The predicted ground configuration had a very poor 10-dB impedance matching at 6.25 GHz with a discontinuity in the in-band performance. With implementation of the vias, the proposed ground configuration asserted wideband performance due to the balanced reference signals at the corresponding antenna ports. The conducting pad at the top is also an integral part of the EM surface wave trapments. The isolation coefficients are shown in Figure 15b, in which very poor isolation of −10 dB magnitudes was observed in the predicted ground configuration, and the isolation was effectively improved with a magnitude of >−20 dB for the proposed ground configuration, reported here.

Figure 15a shows the effect of S11, dB on the variation of diameter of metallic vias. When the vias’ diameters were kept at 0.1 mm, the wideband characteristics were observed with <−15 dB matching response. Similarly, when the vias’ diameters were kept at 0.3 mm, the lower bands and upper bands shrank. So, the proposed via diameter was finally kept at 0.2 mm to yield the desired in-band performance, specifically required from the application perspective.

## 5. Equivalent Circuit Model

**Figure 16:**
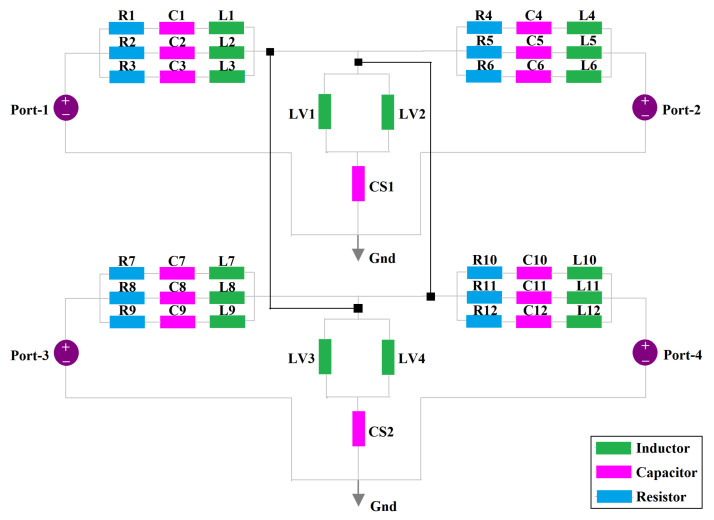
The equivalent circuit model (ECM) of the proposed four-element antenna configuration. The equivalent circuit parameters are: R1 = R4 = R7 = R10 = 59.35 Ω, R2 = R5 = R8 = R11 = 37.36 Ω, R3 = R6 = R9 = R12 = 89.11 Ω, C1 = C4 = C7 = C10 = 0.157 pF, C2 = C5 = C8 = C11 = 0.023 pF, C3 = C6 = C9 = C12 = 0.1017 pF, L1 = L4 = L7 = L10 = 6.98 nH, L2 = L5 = L8 = L11 = 15.3 nH, L3 = L6 = L9 = L12 = 4.78 nH, LV1 = LV2 = LV3 = LV4 = 0.25 nH, CS1 = CS2 = 1.5 pF, Z${}_{0}$ = 50-Ω.

**Figure 17:**
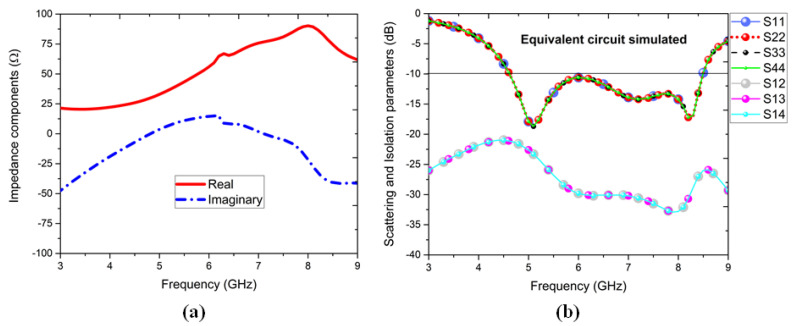
(**a**) Impedance parameters and (**b**) equivalent circuit model (ECM) scattering and isolation coefficients of the four-element antenna configuration.

An equivalent circuit model was designed and simulated in the Advanced Design System (ADS), and the circuit model is shown in Figure 16 with its circuit parameters. The impedance components are shown in Figure 17a, in which real and imaginary impedances exhibit parallel resonances; henceforth, the circuit model can be asserted with parallel-fed series RL-C components. Each antenna consists of independent RL-C components owing to the symmetry, excited by an impedance terminal of Z${}_{0}$ = 50-Ω. So, the collective R-LC components are integrated through inductive metallic vias (LV1, ... , LV4) and dielectric capacitances (CS1, CS2). Each antenna’s components are cross-coupled via interconnective nodes to connect port-1 to port-2, 3, and 4 and the same for all other corresponding ports for the antenna port symmetry and circuit integrity. Hence, the circuit parameters are predicted and calculated by the formulas reported [38,51,52]. The equivalent circuit simulated results for a four-element antenna are highlighted in Figure 17b, which resembles well the full-wave simulated outcomes. Through an equivalent circuit model (ECM), the analysis carried for the proposed four-element through frequency domain analysis was validated.

## 6. Experimental Study

**Figure 18:**
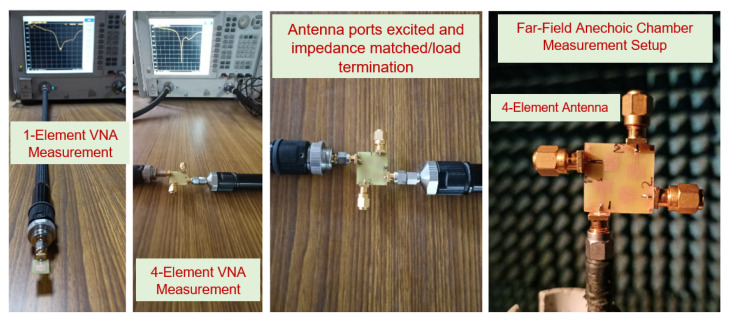
Experimental (measurement) setup of the fabricated one-element antenna and four-element antenna.

The proposed fabricated prototype was validated on a KEYSIGHT N5247A Vector Network Analyzer (VNA), from which the scattering (i.e., one-element and four-element antenna) and isolation parameters (four-element antenna) were extracted. During the measurement process, port-1 was excited and port-2, 3, and 4 were impedance matched/terminated by 50-Ω loads, as illustrated in Figure 18.

### 6.1. S-Parameters and Antenna Gain

**Figure 19:**
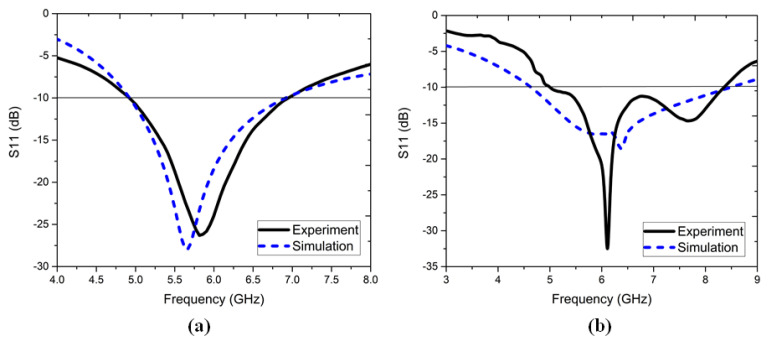
Simulated and experimental results of S11, dB: (**a**) one-element antenna and (**b**) four-element antenna configurations.

**Figure 20:**
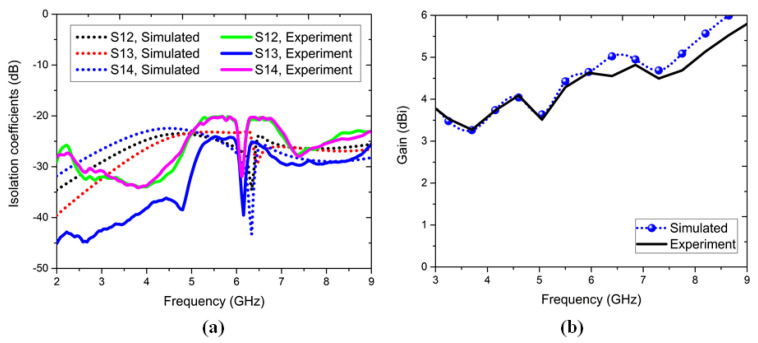
Simulated and experimental results of the (**a**) four-element antenna isolation coefficients and (**b**) four-element antenna gain.

The simulated and experiment results of the S11, dB plots for the one-element antenna and four-element antenna are shown in Figure 19. The one-element antenna had good agreement with the experiment results with slight drift at the resonant frequency band. Similarly, the four-element antenna showed an experiment response from 4.9–8.5 GHz with 53.73% IBW. The isolation coefficients of the simulated and experiment results are plotted in Figure 20a, with the measured isolation magnitude > 21 dB in the operating band. The gain plots for the simulated 4–5.5 dBi and experimental 4–5 dBi results are shown in Figure 20b. Here, the experimental results’ variations were attributed to the substrate quality, connector losses, and tri-port 50-Ω matched load termination effects.

### 6.2. Radiation and Efficiency Performance

**Figure 21:**
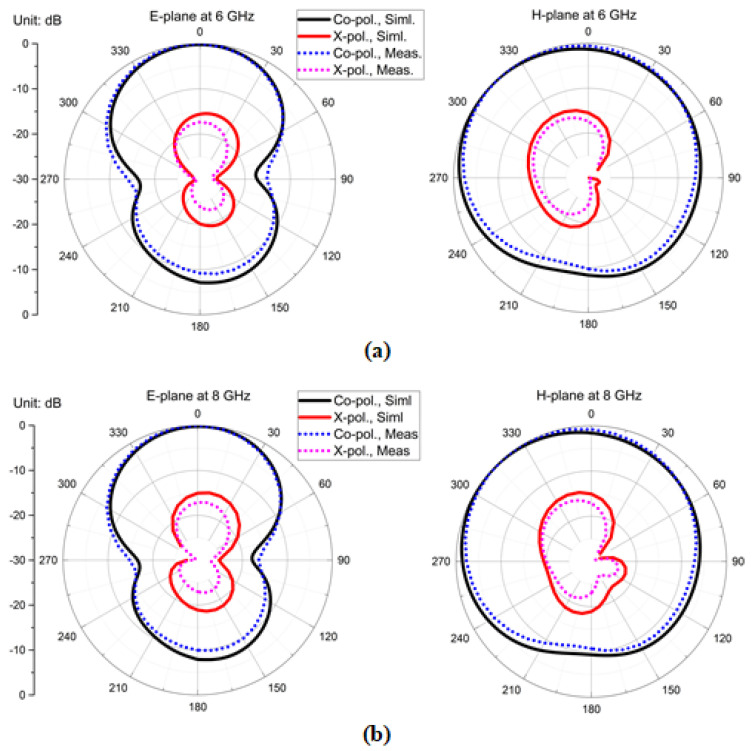
Simulated and measurement patterns for the four-element antenna configuration: (**a**) E-and H-plane at 6 GHz and (**b**) E-and H-plane at 8 GHz, respectively.

**Figure 22:**
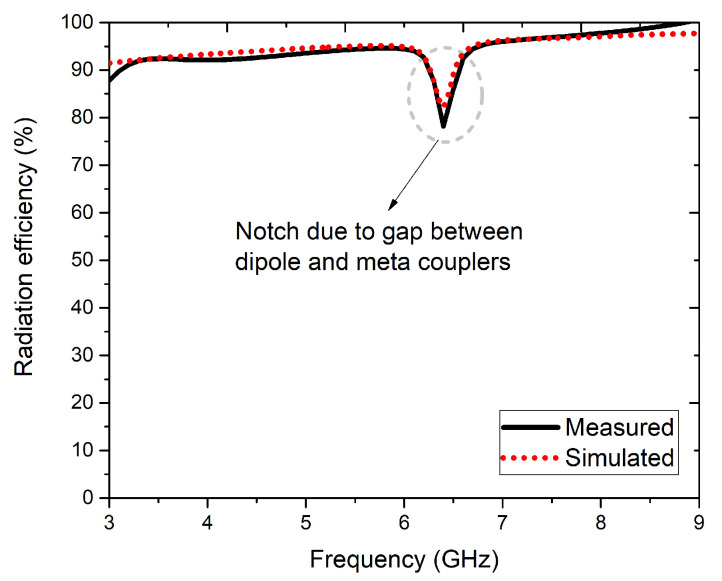
Simulated and measured radiation efficiency plot of the four-element antenna configuration.

Far-field radiation patterns at *f* = 6 GHz and *f* = 8 GHz for both principal radiations at the E-and H-planes were measured in the anechoic chamber, where the co-pol. and X-pol. magnitudes were traced. Figure 21a highlights the far-field radiation patterns at 6 GHz; the E-plane co-pol. magnitudes had figure-of-eight/dipole patterns with X-pol. magnitude < −15 dB, and the H-plane co-pol. magnitudes had omni-directional pattern with X-pol. magnitude < −18 dB. Similarly, Figure 21b presents the far-field radiation patterns at 8 GHz, the E-plane co-pol. magnitudes has figure-of-eight/dipole patterns with X-pol. magnitude < −18 dB and the H-plane co-pol. magnitudes had omni-directional patterns with X-pol. magnitude < −20 dB. The simulated and measured radiation efficiency plot is shown in Figure 22, and the notch effect at 6.25 GHz was due to the gap between the dipole element and meta-coupler. Here, the radiation efficiency was >90% in the operating band and was around 80% at 6.25 GHz.

### 6.3. Diversity Performance

**Figure 23:**
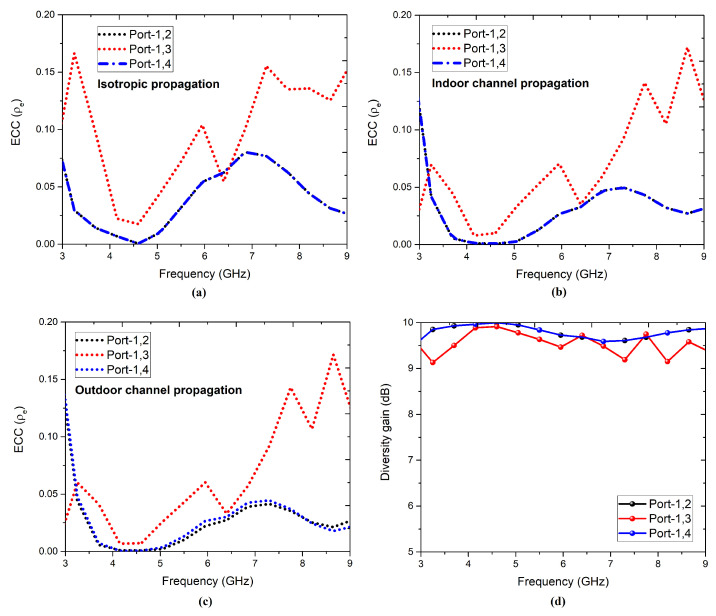
(**a**) ECC for isotropic, (**b**) ECC for indoor, and (**c**) ECC for outdoor environments (propagation), and (**d**) diversity gain of the four-element antenna configuration.

As shown in Figure 23, the diversity performance was evaluated through the envelope channel coefficient (ECC) using the far-field calculation method [58,59,60,61,62] for better accuracy. During the evaluation, we considered gaussian for elevation and uniform for azimuth as the angular power density function. For an isotropic propagation, the cross-pol discrimination (XPD = 0 dB), in which ECC was <0.165 at all ports with respect to port-1. For the indoor propagation, we evaluated with a horizontal/vertical mean = 5 and horizontal/vertical variance = 20, with an ECC < 0.16 at all ports with respect to port-1. For the outdoor propagation, we evaluated with a horizontal/vertical mean = 10 and horizontal/vertical variance = 20, with an ECC < 0.16 at all ports with respect to port-1. Hence, in the different environments, the evaluated ECC (far-field) was <0.165 in the operating band, which shows its potential for diversity applications. The diversity gain (DG) evaluated from ECC (isotropic environment) was found to be >9 dB in the operating spectrum.

**Figure 24:**
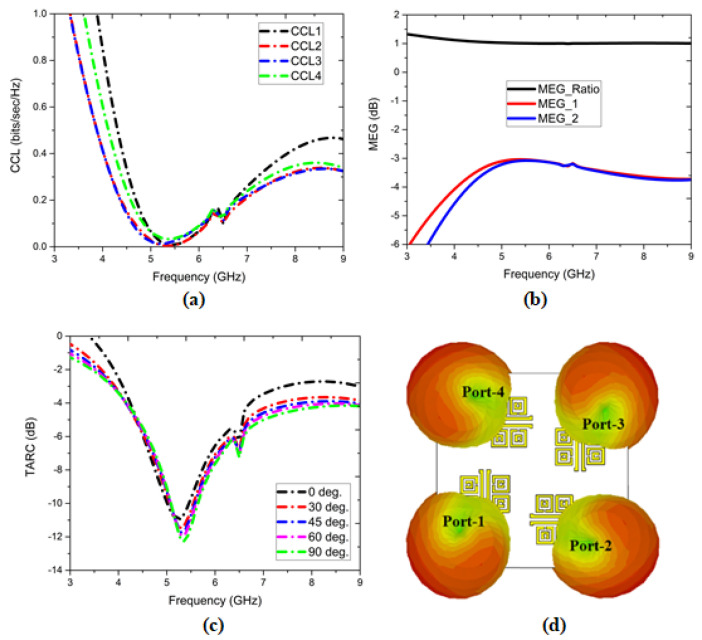
(**a**) Channel capacity loss (CCL), (**b**) mean effective gain (MEG), (**c**) total active reflection coefficient (TARC), and (**d**) 3D far-field radiation pattern of the four-element antenna configuration.

The channel capacity loss (CCL) was calculated from the channel matrix method and was evaluated for each antenna port. Thus, the CCL was <0.425 for all antenna ports in the operating band, highlighted in Figure 24a. The mean effective gain (MEG) was evaluated for port-1 with respect to port-2, 3, and 4 and port-2 with respect to port-1, 3, 4 for brevity (as each port exhibits symmetry in the MIMO configuration) in which the magnitude components were <3 dB, and the ratio was ≅1 as shown in Figure 24b, confirming its wideband behavior.

**Figure 25:**
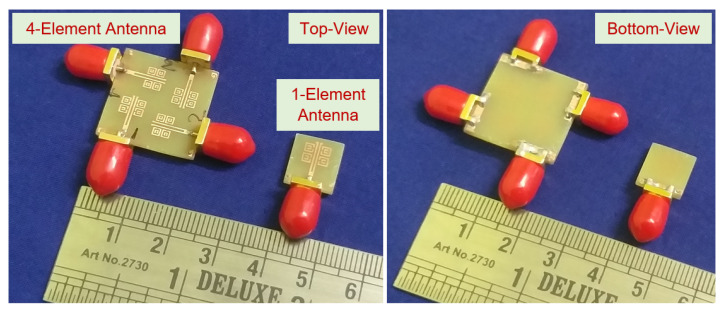
The fabricated prototypes of the one-element antenna and four-element antenna configurations with their alternate views.

The total active reflection coefficient (TARC) [63,64,65] for different angles swept from (0°, ... , 90°) were evaluated, and the reflection magnitudes were insensitive to angle variations. The TARC plot is shown in Figure 24c. The 3D far-field radiation patterns at *f* = 5.8 GHz are shown in Figure 24d. The compactness of the antenna is highlighted in Figure 25 depicting the fabricated prototypes of the one-element antenna and four-element antenna configurations.

Table 1 highlights the comparison analysis of the different four-element antennas with performance metrics. It is observed that the proposed antenna accommodated four-antenna elements and occupied a miniaturized footprint. The antenna asserted good wideband 10-dB impedance response, and the configuration had no external decoupling network to achieve isolation. By using simple dipole elements and meta-couplers, the radiators achieved design miniaturization and the metallic vias integrated into the ground improved the overall bandwidth and good isolation responses at the corresponding ports. The metallic vias, assembled to the conducting pads via a ground, kept each antenna port signal on the same voltage reference plane. The meta-couplers acted as a dual element, an inductive coupler and restored EM-surface waves to be trapped into the nested loop structures, thus, also as a decoupler. Hence, the diversity metrics evaluated showed its potential in a low correlation coefficient for isotropic, indoor, and outdoor channel environments with an average ECC (far–field) < 0.165 in the operating bands. So, the diversity gain with >9 dB computed from ECC components had the ability to multiplex correlate and uncorrelated signals. The MEG ratio almost converged to unity showing the wideband behavior to be exploited in RF wireless environments.

## 7. Conclusions

In this research article, a four-element antenna was designed and investigated, using a simple dipole element and CSRR meta-couplers, where the proposed antenna achieved miniaturization. By utilizing the concept of the integration of metallic vias into the ground plane, the 10-dB impedance matching was quite improved and asserted a wideband response with improved isolation characteristics, without using any decoupling network mechanism. The proposed four-element antenna operates from (4.6 to 8.6) GHz with 60.6% IBW and the isolation coefficients of >−20 dB. The diversity parameter showed good attributes in indoor and outdoor environments with low ECC and good diversity gain. Here, the far-field patterns had a good and stable radiation gain of 4–5.5 dBi with excellent radiation efficiency of 80–90%. The simulated and measured performances were in good agreement, as shown in Table 1, and can be potentially explored in wireless RF communication systems and 5G applications.

## Figures and Tables

**Table 1:** The performance comparison of the four-element antenna configuration over the reported antenna designs in [12,13,14,15,16,17,18,19,20,21,22,23].

Ref.	Antenna	Operation	N	Isolation	Gain	Radiation	ECC
	Size	(GHz)		(dB)	(dBi)	Eff. (%)	
[12]	2.5λo × 1.2λ_o_	(3.4–5)	4	−16.5	(4.9–5.1)	85	0.01
[13]	2π × (1.3λ_o_)	(3.3–5)	4	−13	—	86	0.05
[14]	2.2λo × 1.5λ_o_	Multibands	4	−16	5	70	0.5
[15]	2.3λo × 1.1λ_o_	Multibands	4	−15	(1.3–4.5)	(70–80)	0.2
[16]	2.2λo × 2.2λ_o_	(1.55–6)	4	−16	(8–10)	84	0.025
[17]	0.5λo × 0.6λ_o_	(3–10)	4	−20	—	(65–70)	0.0025
[18]	1.9λo × 1.9λ_o_	(5.15–5.35)	4	−20	4.9	89	0.11
[19]	1.5λo × 1.4λ_o_	Multibands	4	−19	(3–3.5)	—	0.05
[20]	1.4λo × 0.4λ_o_	(3.5–6.8)	4	−15	—	—	—
[21]	0.7λo × 0.7λ_o_	Multibands	4	−19	(2.7–5.1)	—	0.007
[22]	0.8λo × 0.8λ_o_	Multibands	4	−16	4	85	0.025
[23]	0.6λo × 0.5λ_o_	(3.2–5.85)	4	−17.5	3.5	85	0.05
Proposed	0.3λo × 0.3λ_o_	(4.6–8.6)	4	>−20	(4–5.5)	90%	0.165

**Notations Used-Ref**: references used, **N**: number of radiating elements, maximum value of isolation is considered for all the cases, **Radiation Eff.**: radiation efficiency, and **ECC**: envelope correlation coefficient λ_o_ is the operating wavelength at 5 GHz.

## Data Availability

Not applicable.
